# The association between multiple trajectories of macronutrient intake and the risk of new‐onset diabetes in Chinese adults

**DOI:** 10.1111/1753-0407.13555

**Published:** 2024-05-09

**Authors:** Sizhe Wang, Guo Ruirui, Xiaotong Li, Fengdan Wang, Zibo Wu, Yan Liu, Yibo Dong, Bo Li

**Affiliations:** ^1^ Department of Epidemiology and Biostatistics, School of Public Health Jilin University Changchun China

**Keywords:** macronutrient, multitrajectories, new‐onset diabetes, prospective study

## Abstract

**Background:**

The association between macronutrient intake and diabetes is unclear. We used data from the China Health and Nutrition Survey to explore the association between macronutrient intake trajectories and diabetes risk in this study.

**Methods:**

We included 6755 participants who did not have diabetes at baseline and participated in at least three surveys. The energy supply ratio of carbohydrate, protein, and fat was further calculated from dietary data; different macronutrient trajectories were determined using multitrajectory models; and multiple Cox regression models were used to evaluate the association between these trajectories and diabetes.

**Results:**

We found three multitrajectories: decreased low carbohydrate‐increased moderate protein‐increased high fat (DLC‐IMP‐IHF), decreased high carbohydrate‐moderate protein‐increased low fat (DHC‐MP‐ILF), and balanced‐macronutrients (BM). Compared to the BM trajectory, DHC‐MP‐ILF trajectories were significantly associated with increased risk of diabetes (hazard ratio [HR]: 3.228, 95% confidence interval [CI]: 1.571–6.632), whereas no association between DLC‐IMP‐IHF trajectories and diabetes was found in our study (HR: 0.699, 95% CI: 0.351–1.392).

**Conclusions:**

The downward trend of high carbohydrate and the increasing trend of low fat increased the risk of diabetes in Chinese adults.

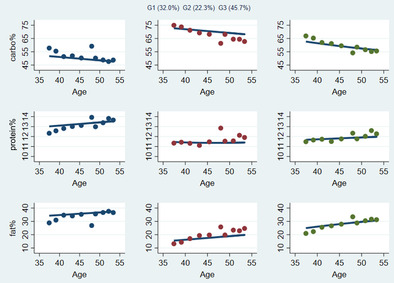

## Graphical abstract

Highlights

- This study explored the multitrajectories of macronutrients in the Chinese adult population over a period of 26 years. The impact of each macronutrient ratio on diabetes was comprehensively considered. More ideas are provided for the prevention of diabetes.

## 1 INTRODUCTION

Diabetes is a common chronic disease worldwide. Over the past few decades, the prevalence of diabetes has increased significantly, becoming a “growing epidemic”.^1^ The International Diabetes Federation estimated that 463 million participants aged 20 and older had diabetes worldwide in 2019, and on this trend, the number was expected to increase to 700.2 million by 2045.^2^ What is more, diabetes has the second most negative effect on reducing health‐adjusted life expectancy worldwide.^3^ It poses a serious health threat and a heavy economic burden. Total global diabetes‐related health expenditures for adults aged 20–79 years were estimated at $760 billion in 2019 and were expected to grow to $825 billion per year by 2030.^2^

Among the many factors influencing diabetes, diet, as a modifiable factor, has a complex association with diabetes. Macronutrients – carbohydrate, protein, and fat – provide energy and essential components for sustaining life. Overall, the proportion of macronutrient intake is inextricably linked to health.^4^ The macronutrient composition of the diet has been shown to play an important role in glucose metabolism and insulin secretion in healthy adults and subjects with Type 2 diabetes Mellitus (T2DM).^5^ However, studies on this topic have yielded inconclusive results. One study showed that higher carbohydrate raised Glycemic Index values, and higher protein lowered these values, but the results on fat were not significant.^6^ In addition, a 2‐year multicenter randomized controlled trial found that a low‐fat, high‐protein diet was not superior to a low‐fat, high‐carbohydrate diet in terms of glycemic control in patients with T2DM.^7^

Most studies on macronutrients and diabetes considered only one or two of the three components(carbohydrate, protein, and fat)^8^, ^9^; however, there was a well‐known “seesaw effect” in the study of macronutrients: when the intake of one macronutrient (such as carbohydrate) rose, the energy contribution of other macronutrients (such as fat and protein) must fall relatively.^10^ Therefore, it was more realistic to consider the three as a whole. In addition, previous studies have more often explored macronutrient intake over shorter periods of time and were rarely large prospective cohort studies.^11^ These studies did not consider changes in macronutrient intake over time. Considering chronic diseases like diabetes, the development cycle was 10 years or more.^10^ In this regard, there were no long‐term data demonstrating the effect of different macronutrient ratios. More important, no studies have reported the association between macronutrient trajectory development and diabetes in the Chinese population.

Accordingly, on the basis of the data from the 1989–2015 China Health and Nutrition Survey (CHNS), our study used a group‐based multitrajectory model to explore the association between multitrajectories of macronutrients and diabetes and provided more ideas for further diabetes prevention through diet.

## 2 MATERIALS AND METHODS

### 2.1 Study population

**FIGURE 1:**
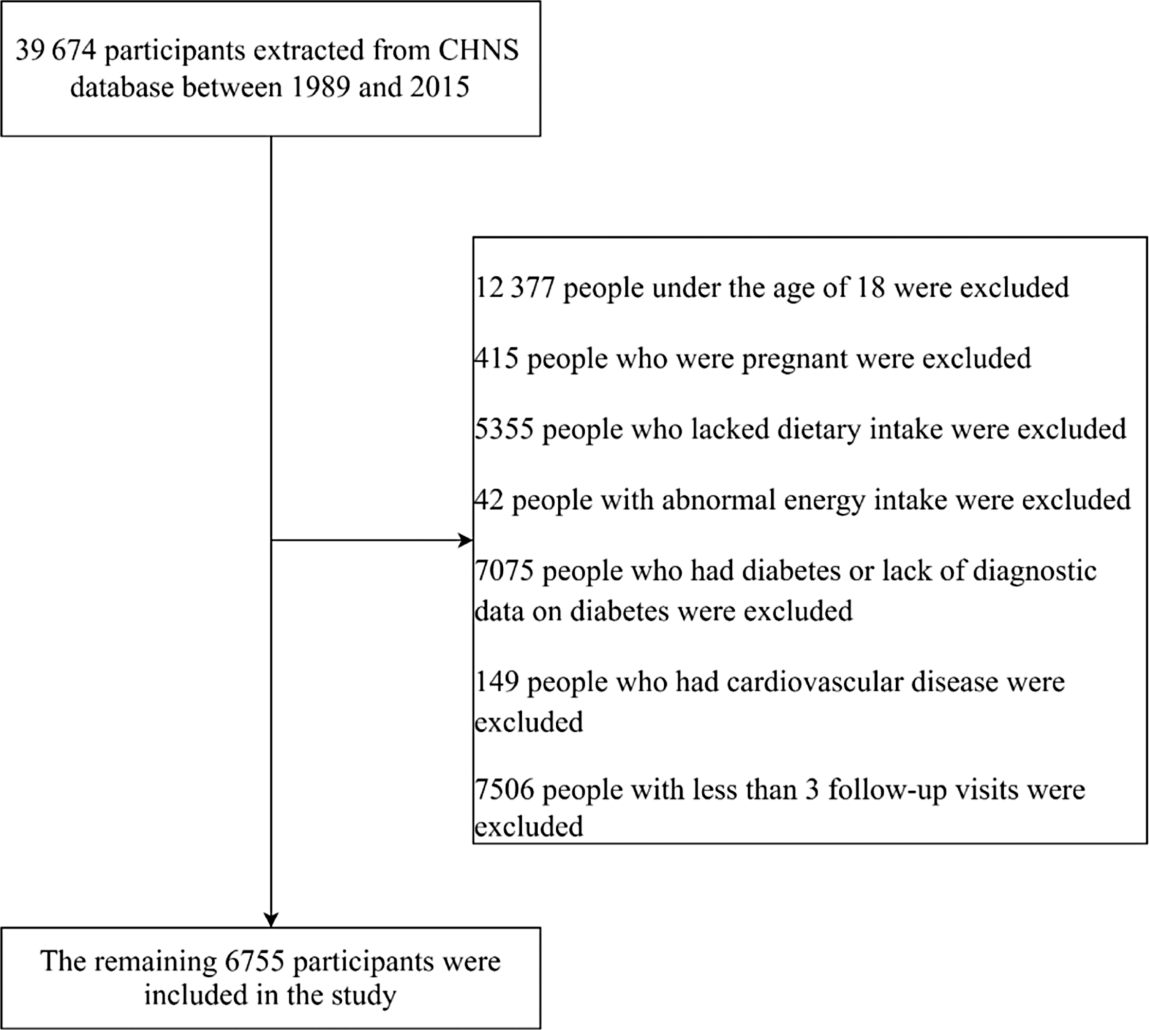
Flow chart for inclusion of participants in the screening process. CHNS, China Health and Nutrition Survey.

CHNS is an ongoing, multilevel, prospective survey based on the Chinese population, which contained nine provinces and three autonomous regions. Since 1989, 10 waves of surveys have been conducted (1989, 1991, 1993, 1997, 2000, 2004, 2006, 2009, 2015, and 2018). The specific content has been described in detail elsewhere.^12^ Because the data for 2018 were not yet released, in this analysis, the data were based on the 10 waves of CHNS conducted between 1989 and 2015. Up to 2015, 39 674 participants have completed the survey. We excluded participants with the following characteristics: participants under the age of 18, participants who were pregnant, participants who did not have a dietary survey, participants with abnormal energy intake (>5000 or <500 kcal/day), participants who lacked diagnostic data on diabetes or had diabetes, and participants who had cardiovascular diseases at the time of the first survey. Given that the multitrajectory model required at least three surveys, we further excluded participants with less than three waves of investigation. Finally, 6755 participants over the age of 18 were included in this study (Figure 1).

CHNS has been approved by the Institutional Review Board of the University of North Carolina at Chapel Hill and the National Institute of Nutrition and Food Safety of the Chinese Center for Disease Control and Prevention. All participants provided written informed consent.

### 2.2 Exposure variable

Dietary intake was assessed using individual‐level dietary recall data: 24‐h dietary recall for 3 consecutive days, including food diaries and food photographs, and household‐level food consumption data: the percentage of oils and condiments in the household inventory consumed by each member was calculated proportionally for the same 3 days, converting household survey data to individual intake.^13^ Data were collected by professional trained dietitians. Detailed descriptions of dietary assessments were available elsewhere.^14^ The per capita daily nutrient intake was further calculated according to the China Food Composition Table. Researchers calculated the average daily nutrient intake of the average person, including total energy, carbohydrate, protein, and fat.

In this study, we further calculated the percentage of energy from carbohydrate, protein, and fat. The formula was carbohydrate percentage of energy intake (% E) = ([carbohydrate (g) × 4]/total energy intake [kcal] × 100), protein percentage of energy intake (% E) = ([protein (g) × 4]/total energy intake [kcal] × 100), fat percentage of energy intake (% E) = ([fat (g) × 9]/total energy intake [kcal] × 100).

### 2.3 Outcome measures

The diagnosis of diabetes was determined based on a questionnaire administered to the participants at each follow‐up visit. For the question “Has the doctor told you that you have diabetes?” A “yes” answer to this question was defined as diabetes. In addition, blood samples were collected and measured only in 2009. Therefore, an additional criterion (fasting glucose ≥7.0 mmol/L or glycated hemoglobin [HbA1c] ≥6.5%)^15^ was added in 2009 to confirm the disease.

### 2.4 Covariates

The following variables were collected through questionnaires: age; sex (men and women); educational level (middle school and below, high school and above); geographic region (urban and rural); annual per capita household income (inflated to the value of the 2015); smoking status (yes or no); alcohol drinking (yes or no); total energy intake; waist circumference (WC); body mass index (BMI: weight [kg] divided by height squared [m^2^]); hypertension (self‐reported hypertension or systolic blood pressure ≥ 140 mmHg or diastolic blood pressure ≥ 90 mmHg); and cardiovascular disease (myocardial infarction or stroke) (yes or no). We recorded time for physical activities (PA) including family activities, career activities, traffic activities, and leisure activities, then calculated the total metabolic equivalent of PA (metabolic equivalents/week) according to the Compendium of Physical Activities.^16^ Sedentary time (ST) was the sum of television, computer, reading, and rest time. For all covariates, we used the baseline measurements.

### 2.5 Statistical analysis

Quantitative variables were described by mean ± SDs, the difference between groups was compared by Student's *t* test or analysis of variance. Categorical variables were expressed as numbers (percentage) and the chi‐square test was used for comparisons between groups.

To examine the association between changes in macronutrient development and diabetes over time, we used group‐based multitrajectory modeling to determine subgroups by identifying the percentage of energy from carbohydrate, protein, and fat. The multitrajectory model was an extension of the group‐based trajectory model that defined different groups according to multiple metrics instead of one and was fitted using the “traj” package in Stata.^17^ In order to determine the optimal number of groups, we first set the order of the polynomial function of the trajectory group to 3, fitted two to five groups respectively, and selected the optimal number of groups using the following criteria: (a) minimum Bayesian information criterion; (b) average posterior allocation probability > 0.70; (c) entropy > 0.80; and (d) proportions of participants in each trajectory group ≥5%. More detailed parameters were shown in Table S1. After determining the number of groups, the polynomial order of each trajectory was chosen based on statistical significance.

Cox proportional risk model was used to examine the association between different trajectories of macronutrients and new‐onset diabetes (expressed as risk ratios [HR] with 95% confidence intervals [CI]). Model 1 adjusted for age, sex, education, income, geographic region, BMI, and WC. Model 2 further adjusted for smoking, alcohol consumption, PA, and ST. Model 3 further adjusted for hypertension and total energy intake. SPSS 24.0 and STATA 17.0 were used for statistical analysis. A two‐sided *p* value of <.05 was considered statistically significant.

## 3 RESULTS

### 3.1 Participant characteristics

Table 1 showed the baseline characteristics of the group by investigator with new‐onset diabetes. A total of 6755 adult participants were included in the study. During the period of follow‐up, a total of 170 cases of diabetes were identified, with a mean age of 45.45 ± 12.28 years (labeled as “diabetes group”). The mean age of participants in the “no diabetes group” was 38.84 ± 12.95 years. Age, the proportion of urban population, BMI, WC, total energy intake, and prevalence of hypertension were significantly higher in the diabetes group than in the no diabetes group (*p* < .05). ST was lower than in the no diabetes group (*p* < .05), no significant differences in other aspects (*p* > .05).

**TABLE 1:** Baseline characteristics of investigators in the diabetes and no diabetes groups.

Variables	Diabetes (*n* = 170)	No diabetes (*n* = 6585)	*P* value
Age (years)	45.45 ± 12.28	38.84 ± 12.95	<.001
Sex (%)			.541
Men	75 (44.12)	3061 (46.48)	
Women	95 (55.88)	3524 (53.52)	
Geographic region (%)			.002
Urban	76 (44.71)	2183 (33.15)	
Rural	94 (55.29)	4402 (66.85)	
Education level (%)^a^			.239
Middle school and below	128 (75.29)	5195 (78.89)	
High school and above	39 (22.94)	1273 (19.33)	
Smoking (%)^a^			.899
Yes	43 (25.30)	1539 (23.37)	
No	89 (52.35)	3110 (47.23)	
Drinking (%)^a^			.408
Yes	53 (31.18)	1680 (25.51)	
No	80 (47.06)	2942 (44.68)	
Physical activity (metabolic equivalents/week)	48.80 ± 72.90	53.13 ± 84.03	.505
Sedentary time (hours/week)	7.55 ± 34.71	19.97 ± 64.71	<.001
Body mass index (kg/m^2^)^a^	22.39 ± 3.31	22.08 ± 2.88	<.001
Waist circumference (cm)^a^	85.80 ± 9.72	78.53 ± 9.63	<.001
Annual per capita household income (yuan)^a^	4596.58 ± 5112.33	4853.78 ± 5441.41	.579
Hypertension (%)^a^			<.001
Yes	38 (22.35)	542 (8.23)	
No	95 (55.88)	4111 (62.43)	
Total energy (kcal/d)	2669.45 ± 857.58	2490.86 ± 699.91	<.001
Carbohydrate (%)	62.37 ± 11.17	63.38 ± 12.41	.293
Protein (%)	12.32 ± 2.29	11.93 ± 2.51	.045
Fat (%)	25.03 ± 10.41	23.97 ± 11.61	.238

^a^ Missing data.

### 3.2 Multitrajectory of the percentage of energy supplied by macronutrients

**FIGURE 2:**
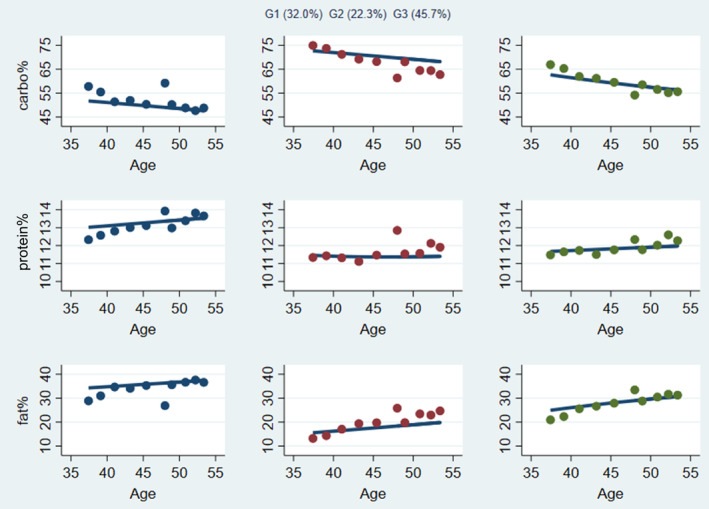
Multitrajectories of macronutrients (carbohydrate%, protein%, and fat%).

In the sample of CHNS data from 1989 to 2015, based on the results of group‐based trajectory modeling analysis, we finally selected three trajectories on carbohydrate, protein, and fat energy intake percentage (Figure 2). The trajectories were named after the standard ranges of carbohydrate intake of 50%–65%, protein intake of 10%–15%, and fat intake of 20%–30% in Chinese Dietary Reference Intakes (2013).

The characteristics of the trajectories were shown in Figure 2. The first group, which included 32.0% of the participants, was characterized by a carbohydrate energy percentage of <50.0% and decreasing over time. The percentage of energy supply of protein was high within the normal range. The percentage of energy from fat was about 35.0%, which increased slowly over time. This group was therefore called decreased low carbohydrate (DLC), increased moderate protein (IMP), and increased high fat (IHF) (DLC‐IMP‐IHF). The second group, consisting of 22.3% of the participants, was characterized by a percentage of energy supplied in carbohydrate of about 75.0%, which was slowly declining but still >65%. The percentage of energy supply from protein was in the middle level between 11.0% and 12.0%. The percentage of energy from fat was about 15.0%–20.0%, which tended to increase slightly over time. The group was described as decreased high carbohydrate (DHC), moderate protein (MP), and increased low fat (ILF) (DHC‐MP‐ILF). The third group contained 45.7% of the participants and was characterized by a reduction in the percentage of energy supplied in carbohydrate from approximately 65.0% to 55.0%. The percentage of energy supply from protein was around 12.0%. The percentage of energy supply from fat gradually approached 30.0%. We referred to this group as balanced macronutrients (BM).

### 3.3 Baseline characteristics of different macronutrient trajectories

Among these three trajectory groups, compared with the other two groups, the DLC‐IMP‐IHF group had the lowest mean age; the highest educational level, BMI, income, PA, and ST; and were more likely to be urban residents, nonsmokers, and nonhypertensive patients (*p* < .05). The DHC‐MP‐ILF group had the lowest education and income levels, BMI, PA, and ST and the highest mean age and were more likely to be smokers, rural residents, and hypertension patients (*p* < .05). There were significant differences in total energy, carbohydrate, protein, and fat intake among the three groups (*p* < .05). (Table 2).

**TABLE 2:** Baseline characteristics of multitrajectory study population.

Variables	DLC‐IMP‐IHF (*n* = 2145)	DHC‐MP‐ILF (*n* = 1482)	BM (*n* = 3128)	*P* value
Age (years)	34.79 ± 11.45	44.39 ± 12.92	39.35 ± 12.99	<.001
Sex (%)				.319
Men	976 (45.50)	677 (45.68)	1483 (47.41)	
Women	1169 (54.50)	805 (54.32)	1645 (52.59)	
Geographic region (%)				<.001
Urban	1209 (56.36)	202 (13.63)	848 (27.11)	
Rural	936 (43.64)	1280 (86.37)	2280 (72.89)	
Education level (%)^a^				<.001
Middle school and below	1363 (63.54)	1359 (91.70)	2601 (83.15)	
High school and above	760 (35.43)	81 (5.47)	471 (15.06)	
Smoking (%)^a^				<.001
Yes	475 (22.14)	351 (23.68)	756 (24.17)	
No	1170 (54.55)	638 (43.05)	1391 (44.47)	
Drinking (%)^a^				.115
Yes	625 (29.14)	338 (22.81)	770 (24.62)	
No	1007 (46.94)	643 (43.39)	1372 (43.86)	
PA (METs/week)	62.62 ± 94.58	45.45 ± 71.82	50.03 ± 80.49	<.001
ST (hours/week)	34.25 ± 85.93	9.76 ± 42.30	14.34 ± 51.52	<.001
BMI (kg/m^2^)^a^	22.34 ± 3.00	21.70 ± 2.73	22.17 ± 2.89	<.001
WC (cm)^a^	78.62 ± 9.87	78.76 ± 9.12	78.72 ± 9.70	.952
Annual per capita household income (yuan)^a^	7181.01 ± 6556.06	2744.73 ± 4010.05	4301.02 ± 4587.09	<.001
Hypertension (%)^a^				<.001
Yes	143 (6.67)	147 (9.92)	290 (9.27)	
No	1503 (70.07)	842 (56.82)	1861 (59.50)	
Total energy (kcal/d)	2403.26 ± 653.96	2666.64 ± 779.21	2477.3 ± 687.06	<.001
Carbohydrate (%)	53.76 ± 10.58	73.60 ± 8.27	65.07 ± 10.30	<.001
Protein (%)	12.89 ± 2.95	11.37 ± 1.92	11.55 ± 2.23	<.001
Fat (%)	32.36 ± 10.24	14.53 ± 7.87	22.74 ± 9.83	<.001

^a^ Missing data. Abbreviations: BM, balanced macronutrients; BMI, body mass index; DHC, decreased high carbohydrate; DLC, decreased low carbohydrate; IHF, increased high fat; ILF, increased low fat; IMP, increased moderate protein; MET, metabolic equivalent; MP, moderate protein; PA, physical activity; ST, sedentary time; WC, waist circumference.

### 3.4 Association between multitrajectories of macronutrients and diabetes

Table 3 showed the association between the different macronutrient trajectories and the risk of diabetes. Compared to the BM trajectory, after adjusting for age, sex, education, income, geographic region, BMI, and waist circumference, DHC‐MP‐ILF trajectory was significantly associated with increased risk of diabetes (HR: 2.948, 95% CI: 1.450–5.994), further adjusted for all relevant covariates, DHC‐MP‐ILF trajectory still was significantly associated with increased risk of diabetes (HR: 3.228, 95% CI: 1.571–6.632), whereas no association between DLC‐IMP‐IHF trajectory and diabetes was found in our study (*p* > .05).

**TABLE 3:** Association between multitrajectories of macronutrients and new‐onset diabetes.

Trajectories	Model 1		Model 2		Model 3	
	HR (95% CI)	*p* value	HR (95% CI)	*p* value	HR (95% CI)	*p* value
BM	1.000 (reference)		1.000 (reference)		1.000 (reference)	
DLC‐IMP‐IHF	0.770 (0.394 to 1.504)	.444	0.717 (0.362 to 1.422)	.314	0.699 (0.351 to 1.392)	.308
DHC‐MP‐ILF	2.948 (1.450 to 5.994)	.003	3.163 (1.539 to 6.499)	.002	3.228 (1.571 to 6.632)	.001

*Note*: Model 1: Adjusted for age, sex, education, income, geographic region, BMI, and waist circumference. Model 2: Further adjusted for smoking, alcohol consumption, PA, ST. Model 3: Further adjusted for hypertension, total energy intake.

Abbreviations: BM, balanced macronutrients; BMI, body mass index; CI, confidence interval; DHC, decreased high carbohydrate; DLC, decreased low carbohydrate; HR, hazard ratio; IHF, increased high fat; ILF, increased low fat; IMP, increased moderate protein; MP, moderate protein; PA, physical activity; ST, sedentary time.

### 3.5 Sensitivity analysis

To reduce potential contamination from participants who were pre‐diabetic at the beginning of the investigation, a sensitivity analysis was performed after excluding participants who self‐reported diabetes at the first follow‐up. The results were consistent with the previous ones, and the specific data were detailed in Table S2.

## 4 DISCUSSION

In our study, using the data of a prospective cohort of Chinese adults over a 26‐year period, we identified three different macronutrient intake trajectories. The DHC‐MP‐ILF group was significantly associated with an increased risk of diabetes compared with the BM group. The DHC‐MP‐ILF group was characterized by a slowly decreasing high carbohydrate intake over time, fluctuating protein within the normal range and gradually increasing low fat intake.

We found that even though carbohydrate intake has decreased over time, it remained above the recommended range, and similarly, fat intake increased but was still below the recommended range. Similarly, over the decades, the diet of Chinese adults has undergone significant changes, characterized by a decrease in the intake of grains and vegetables and an increase in the intake of animal foods.^18^ Consistent with previous studies, we found that the Chinese population as a whole showed a decrease in the proportion of carbohydrate energy intake, an increase in the proportion of fat energy intake, and a stable proportion of protein energy intake in a small range.^19^ In addition, there was a larger part of the population in the normal macronutrient range. Despite the positive trends, important dietary challenges remained. The focus was on improving macronutrient composition and dietary quality.

Given the change in Chinese macronutrients,^20^ and the complex relationship between macronutrients and diabetes,^21^ we further investigated the link between the two. In the Cox regression analysis of macronutrient trajectories and diabetes, we found that the DHC‐MP‐ILF group was significantly associated with an increased risk of diabetes compared with the BM group. Some previous studies have partially supported our conclusions.^22^ In nondiabetic adults, a US study showed that insulin resistance levels increased linearly with increasing carbohydrate, whereas insulin resistance tended to decrease with increasing protein and fat intake,^23^ and T2DM was a common consequence of insulin resistance.^24^ A 12‐year prospective study in South Korea showed that very low fat or very high carbohydrate intake may increase the risk of T2DM.^25^ In diabetic adults, studies have shown greater improvement in HbA1c on low‐carbohydrate, high‐fat diets than on high‐carbohydrate, low‐fat diets^26^ and clinically significant improvements in glycemic control and weight.^27^ Similarly, a randomized controlled trial with both low‐carbohydrate diet and high‐carbohydrate diet reduced HbA1c and fasting glucose. However, the low‐carbohydrate diet achieved greater improvements in lipid levels and glucose stability.^28^ A study of the Chinese population showed that macronutrient intake was associated with obesity, and consistent with this study, the study also selected a balanced macronutrient group as the control group and thought that relatively low carbohydrate and high fat intake may be beneficial in preventing obesity.^19^ Obesity is thought to be the promoter of T2DM,^29^ which indirectly supports the conclusions of this study.

There were several possible mechanisms by which macronutrients cause diabetes. First, the DHC‐MP‐ILF group had the highest energy intake in this study, and excessive energy intake led to obesity. Obesity was believed to be a promoter of T2DM.^29^ The DHC‐MP‐ILF group also had the highest proportion of carbohydrate. Among the many factors that influenced insulin secretion, dietary carbohydrate had the most significant effect. Protein, depending on the composition of amino acids, stimulated the secretion of insulin, but it also caused the secretion of glucagon (which counteracts the breakdown of insulin); dietary fat had little direct effect on insulin.^30^ Total fat intake was not associated with risk of T2DM, but different sources of fatty acids might be differently associated with the risk of T2DM.^31^ So carbohydrate intake was more important in diabetes.^32^ Similarly, the carbohydrate‐insulin model supported this view.^33^ Here was a specific explanation of how carbohydrate affected blood sugar: on the one hand, most of the carbohydrate in food was absorbed into glucose in the blood circulation.^34^ Higher carbohydrate was directly related to increased blood sugar levels after meals.^35^ On the other hand, because a key underlying mechanism of type 2 diabetes was insulin resistance driven in part by chronic hyperglycemia,^36^ as carbohydrate consumption increases, insulin resistance increases in a linear manner.^23^ In addition, high carbohydrate intake was positively correlated with low‐density lipoprotein cholesterol and elevated triglycerides.^37^ Diabetic dyslipidemia was characterized by elevated with low‐density lipoprotein cholesterol and triglycerides.^38^

Although a high‐carbohydrate and low‐fat diet will increase the risk of diabetes, it also should not be pursued to the opposite of extremes. A mouse experiment showed that ketogenic diet（KD） achieved better glycemic control than a high‐fat diet. However, a long‐term KD disrupted lipid metabolism in diabetic mice, leading to liver lipid accumulation and even nonalcoholic fatty liver disease‐like damage.^39^

This study did not find an increased risk of diabetes in the DLC‐IMP‐IHF group. Similarly, Shan et al showed that there was no statistically significant association between low‐carbohydrate, high‐protein, and high‐fat dietary scores and the incidence of T2DM in the overall population or in the normal caloric intake population.^40^ Moreover, the study has shown that a diet low in carbohydrate and high in fat and protein was unlikely to increase the risk of diabetes in a Japanese population.^41^ But this was different from some previous studies. The study has reported that high‐fat diet intake can affect glucose and lipid metabolism, thereby impairing the function of the liver and other major metabolic organs, gradually increasing hyperglycemia, hyperinsulinemia, and progressive insulin resistance.^42^

This study was based on the CHNS database, in which the reliability of self‐reported 24‐h dietary recall method has been demonstrated.^43^ To our knowledge, this is the first study on diabetes in which macronutrient intake trajectories rather than single time points have been used to better determine the long‐term effects of macronutrient changes on diabetes. More important, we complement the results of previous findings in terms of the distribution of macronutrient intake, rather than a single nutrient assessment, which could provide more comprehensive guidance for dietary interventions. However, this study does have some limitations. First, the database lacked important variables such as family history of diabetes and type of diabetes. Second, blood samples were collected only in 2009, which may have resulted in lower than actual prevalence of diabetes in other years. In addition, self‐reported food diaries have recognized limitations, and underreporting energy intake is common. Finally, this article provides only a rough assessment of the impact of trends in macronutrient intake on diabetes. In fact, the type of carbohydrate,^44^ protein,^45^ and fat^46^ can also have a significant effect on blood glucose. Further high‐quality prospective studies with more plausible methods to determine the results could be followed up to confirm the association between macronutrient intake trajectories and diabetes risk.

## 5 CONCLUSION

In conclusion, this study found that the macronutrient trajectories of Chinese population have shown three different trends over the past 2 decades. In addition, our study found that the decreasing trend of high carbohydrate and the increasing trend of low fat can increase the risk of diabetes. Therefore, it was also necessary to strictly control the proportion of macronutrient within the specified range.

## AUTHOR CONTRIBUTIONS

Sizhe Wang, Guo Ruirui, and Xiaotong Li designed the research; Sizhe Wang, Xiaotong Li, and Guo Ruirui conducted literature searches; Sizhe Wang and Guo Ruirui performed the statistics analyses; Sizhe Wang wrote the manuscript; Sizhe Wang, Guo Ruirui, Xiaotong Li, Fengdan Wang, Zibo Wu, Yan Liu, and Yibo Dong attended the manuscript revision. All authors have read and approved the final manuscript.

## FUNDING INFORMATION

This work was supported by the National Natural Science Foundation of China (No.81973129).

## CONFLICT OF INTEREST STATEMENT

The authors declare that there are no conflicts of interest regarding the publication of this paper.

## Supporting information


**Table S1.** Relevant parameters during the fitting of a multi‐trajectory model for macronutrients.
**Table S2.** Sensitivity analysis to exclude the development of diabetes mellitus at the beginning of follow‐up.

## Data Availability

The datasets generated during the current study are available in the CHNS repository, [https://www.cpc.unc.edu/projects/china].
