# Supplementary material for: The association between multiple trajectories of macronutrient intake and the risk of new‐onset diabetes in Chinese adults
Source: J Diabetes. 2024 May 9;16(5):e13555. doi: 10.1111/1753-0407.13555 (PMC11079633; doi:10.1111/1753-0407.13555)
Supplement: Supplementary file 1 — Table S1. Relevant parameters during the fitting of a multi‐trajectory model for macronutrients. Table S2. Sensitivity analysis to exclude the development of diabetes mellitus at the beginning of follow‐up. [file JDB-16-e13555-s001.docx]

**Supplementary Table 1** Relevant parameters during the fitting of a multi-trajectory model for macronutrients

| **Number of groups** | **Probabilities for each group** | | | | | **BIC (N=6755)** | **APPA for each group** | | | | | **Entropy** |
| --- | --- | --- | --- | --- | --- | --- | --- | --- | --- | --- | --- | --- |
|  | Group1 | Group2 | Group3 | Group4 | Group5 |  | Group1 | Group2 | Group3 | Group4 | Group5 |  |
| 2 | 45.15 | 54.85 |  |  |  | -384130.20 | 0.95 | 0.95 |  |  |  | 0.849 |
| 3 | 22.36 | 44.66 | 32.98 |  |  | -381453.87 | 0.92 | 0.90 | 0.93 |  |  | 0.815 |
| 4 | 32.02 | 14.19 | 22.04 | 31.75 |  | -380422.49 | 0.86 | 0.91 | 0.89 | 0.84 |  | 0.777 |
| 5 | 24.76 | 12.15 | 30.55 | 14.88 | 17.67 | -379853.51 | 0.83 | 0.90 | 0.83 | 0.82 | 0.82 | 0.756 |

**Supplementary Table 2** Sensitivity analysis to exclude the development of diabetes mellitus at the beginning of follow-up

| **Trajectories** | **Model 1** | | **Model 2** | | **Model 3** | |
| --- | --- | --- | --- | --- | --- | --- |
|  | **HR (95%CI)** | ***p*-value** | **HR (95%CI)** | ***p*-value** | **HR (95%CI)** | ***p*-value** |
| BM | 1.000(reference) |  | 1.000(reference) |  | 1.000(reference) |  |
| DLC-IMP-IHF | 0.850(0.424,1.706) | 0.648 | 0.787 (0.386,1.604) | 0.509 | 0.772(0.377,1.580) | 0.479 |
| DHC-MP-ILF | 2.862(1.367,5.996) | 0.005 | 2.979 (1.412,6.288) | 0.004 | 3.013(1.427,6.361) | 0.004 |

Model 1: Adjusted for age, sex, education, income, geographic region, BMI, and waist circumference.

Model 2: Further adjustment for smoking, alcohol consumption, PA, ST.

Model 3: Further adjusted for hypertension, total energy intake.
